# An Open Source 3-D Printed Modular Micro-Drive System for Acute Neurophysiology

**DOI:** 10.1371/journal.pone.0094262

**Published:** 2014-04-15

**Authors:** Shaun R. Patel, Kaushik Ghose, Emad N. Eskandar

**Affiliations:** 1 Nayef Al-Rodhan Laboratories, Department of Neurosurgery, Massachusetts General Hospital, Boston, Massachusetts, United States of America; 2 Department of Anatomy and Neurobiology, Boston University School of Medicine, Boston, Massachusetts, United States of America; Nathan Kline Institute and New York University School of Medicine, United States of America

## Abstract

Current, commercial, electrode micro-drives that allow independent positioning of multiple electrodes are expensive. Custom designed solutions developed by individual laboratories require fabrication by experienced machinists working in well equipped machine shops and are therefore difficult to disseminate into widespread use. Here, we present an easy to assemble modular micro-drive system for acute primate neurophysiology (PriED) that utilizes rapid prototyping (3-d printing) and readily available off the shelf-parts. The use of 3-d printed parts drastically reduces the cost of the device, making it available to labs without the resources of sophisticated machine shops. The direct transfer of designs from electronic files to physical parts also gives researchers opportunities to easily modify and implement custom solutions to specific recording needs. We also demonstrate a novel model of data sharing for the scientific community: a publicly available repository of drive designs. Researchers can download the drive part designs from the repository, print, assemble and then use the drives. Importantly, users can upload their modified designs with annotations making them easily available for others to use.

## Introduction

Single-neuronal studies remain the gold-standard of understanding how our brains encode and process information. Since the late 1960s, researchers have been exploring the activity of individual neurons in awake-and-behaving non-human primates to better understand the neural substrates of cognition and behavior [1]. Despite these advancements, the classic method of inserting a single-probe at a time poses a critical bottleneck for understanding the activity of multiple neurons simultaneously – a more biologically realistic framework.

More recently, researchers have developed innovative microdrive solutions that allow for multi-neuron recordings in both acute [2], [3], semi-chronic [4], and chronic preparations [5]. Although these solutions allow for simultaneous multi-neuron and multi-site recordings, they are either expensive to purchase commercially or are custom-built devices that require experienced machinists working in well equipped model shops to build and assemble. Chronically implanted micro-electrode arrays have also come to the fore as a means of simultaneously recording from populations of neurons [6], [7]. Such arrays are typically limited to superficial and reasonably flat parts of cortex, though some companies are starting to provide arrays with longer electrodes to reach deeper structures. Nevertheless, deep brain structures such as the basal ganglia and hippocampus remain out of range of arrays.

Over the last decade, advances in materials and manufacturing technology have enabled researchers to fabricate micro-drives that allow simultaneous recording from multiple sites in different brain regions. Unfortunately, despite this development, much of the technology remains inaccessible to smaller laboratories with limited operating budgets. Many researchers and industry partners have developed custom solutions to address this problem. However, most – if not all – existing designs require experienced machinists, working in well equipped model shops, to build and assemble. In some cases, the intricate nature of parts and designs result in prohibitively high resources using traditional manufacturing methods. This adds greatly to the overall cost and time required to build the drive.

The advent of 3-d printing technology in the the mid 1980s has revolutionized the manufacturing process. The earliest 3-d printers utilized a process known as stereolithography [8]. In this process, a part is built up by depositing successive layers of a photosensitive polymer, and then curing them with an ultraviolet laser. This technology was followed by the development of selective laser sintering [9], whereby a high-powered laser was used to sinter powdered plastics, metals, glass and other materials to create 3-d objects. The most recent, and common, type of printing used today, is known as fused deposition modeling [10]. This system works by actuating an extrusion nozzle which can selectively control the flow of the build material. Most common materials include thermoplastics which are heated through the extruder and harden when cooled. Applications for 3-d printers are numerous but some notable uses are creating parts for space shuttles, printing jewelry using silver and gold, and printing human organs including blood vessels [11]–[15].

Here we describe PriED, a modular electrode positioning system for acute neurophysiology, made using polymer extrusion additive manufacturing. PriED (Printed Electrode Drive, pronounced the same as the English word “pride”) is constructed from custom designed 3-d printed parts, in combination with commercially available steel and brass components. PriED has been designed specifically to advance current micro-drive technology while at the same time remaining extremely inexpensive and easy to construct. The custom plastic parts are printed using a commercial service and the entire drive requires minimal effort and skill to assemble. The use of 3-d printing enables researchers to easily modify designs and electronically share them with colleagues who can in turn easily print the modified devices for their own use. To facilitate this novel channel for sharing recording equipment we have set up a public repository for drive designs accessible on the internet (https://bitbucket.org/multidrive/pried).

## Results

### Costs and Weights

Pricing may vary depending on the material, type of printer/service, and design. Table 1 itemizes the costs of printing the current version of the drive using the service mentioned here. The total cost of a PriED with a complement of 10 towers is $718 for the single base model and $739 for a stacked model. For comparison, an earlier micro-drive designed in the lab and manufactured in the traditional manner by machinists at a university model shop, cost roughly $1,300 and did not include the ability to easily modify, share, or replace any components.

**Table 1:** Sample costs for 3-d printed parts.

Printed Part	Cost per unit^a^	Quantity
Single piece base	$88	1/drive
Stacked base upper	$57	1/drive
Stacked base lower	$52	1/drive
Tower base	$10	1/tower
Bearing	$10	1/tower
Shuttle	$10	1/tower
Tower cap	$13	1/tower
Sleeve	$10	1/tower
Advancer	$10	1/tower

a Jan 2013 cost in USD rounded to nearest dollar. The service used charges $10 minimum per part.

Although the motorized PriED is not discussed in detail here, the PriED was designed to be controlled both manually and by a motor. The additional cost to motorize the PriED is approximately $400/tower: $200 for the stepper motor (Series 0620, Faulhaber) and $200 for the planetary gear reduction (Series 06/1, Faulhaber). For comparison, a similar commercial micro-drive would cost upwards of $30,000.

The weight of the important drive components and assemblies are given in Table 2. The weight of a stacked drive with the full complement of ten towers will be 115.4 g. As comparison, the weight of a Cilux Crist chamber is 4.4 g.

**Table 2:** Weights for 3-d printed parts.

Printed Part	Weight
Single piece base	16.9 g
Stacked base upper	13.7 g
Stacked base lower	9.7 g
Tower	9.2 g

### Electrode Advance Characteristics

**Figure 1:**
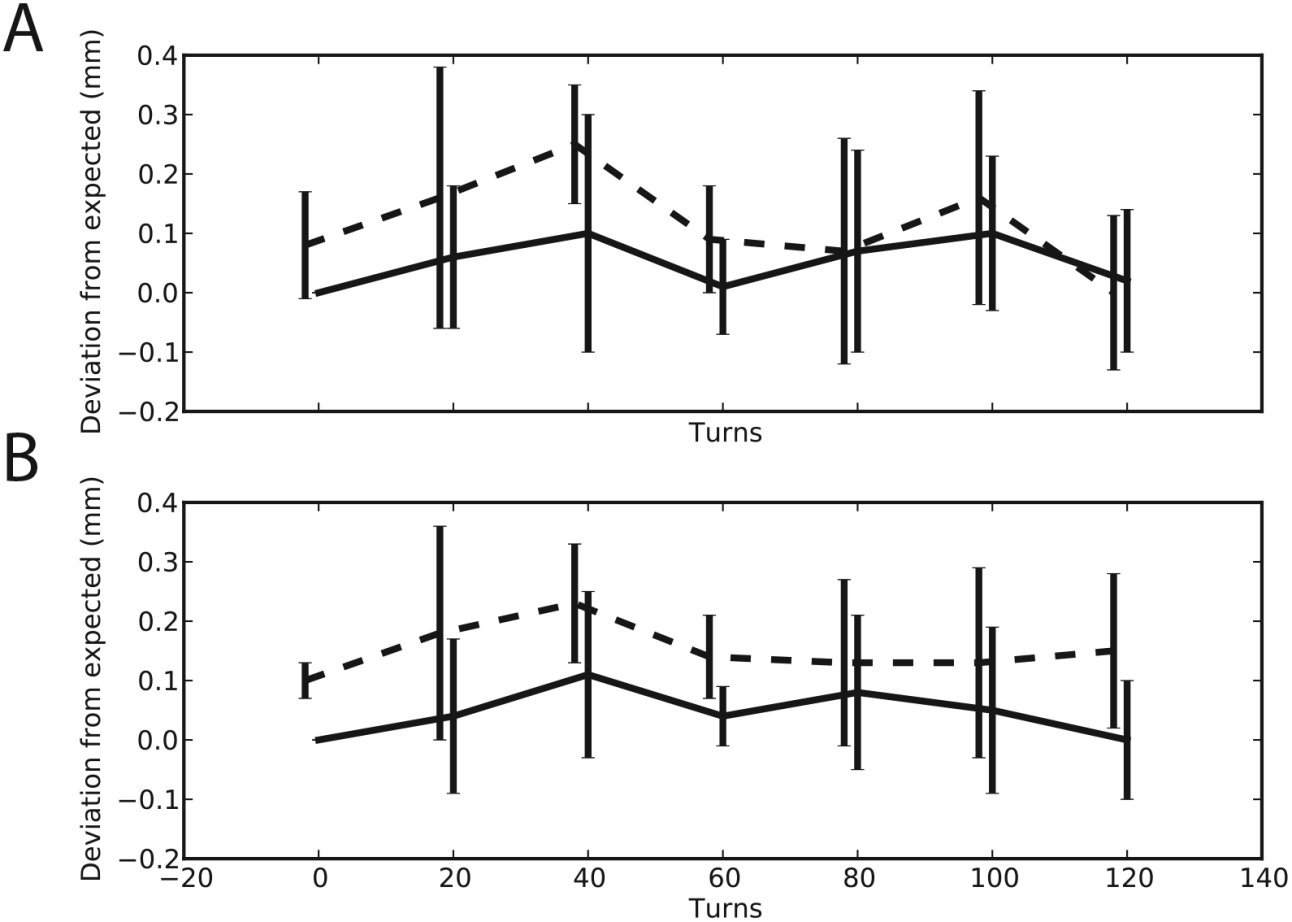
Measurements of electrode advance. advancement and retraction for printed and machined microdrive. A 250 um glass electrode was loaded into the center position of the electrode grid. The drive was placed in rig that allowed us to measure the electrode travel as we turned the advancer. The electrode was advanced and retracted five times. Travel measurements were taken every 20 turns. Solid line shows data from the advancing phase and the dotted line shows data from the retracting phase for the printed (A) and machined version (B) of the microdrive. At the start of each cycle we realigned the electrode position to zero.

Due to the angle between each tower and grid, a natural bow is created in the electrode that could introduce an error between the nominal distance the shuttle travels and the actual distance the electrode advances. We characterized this difference by performing five repeated measurements of lowering and raising the shuttle and computing the difference between the expected and measured distance (Figure 1). We found minimalno deviation in the expected travel distance when advancing the shuttle (within our measurement error). However, we did observe a backlash effect when retracting the shuttle. For comparison, we also tested a similarly designed microdrive that was professionally machined and found minimal differences in electrode advancement and retraction characteristics between the two versions.

### Recording Stability

**Figure 2:**
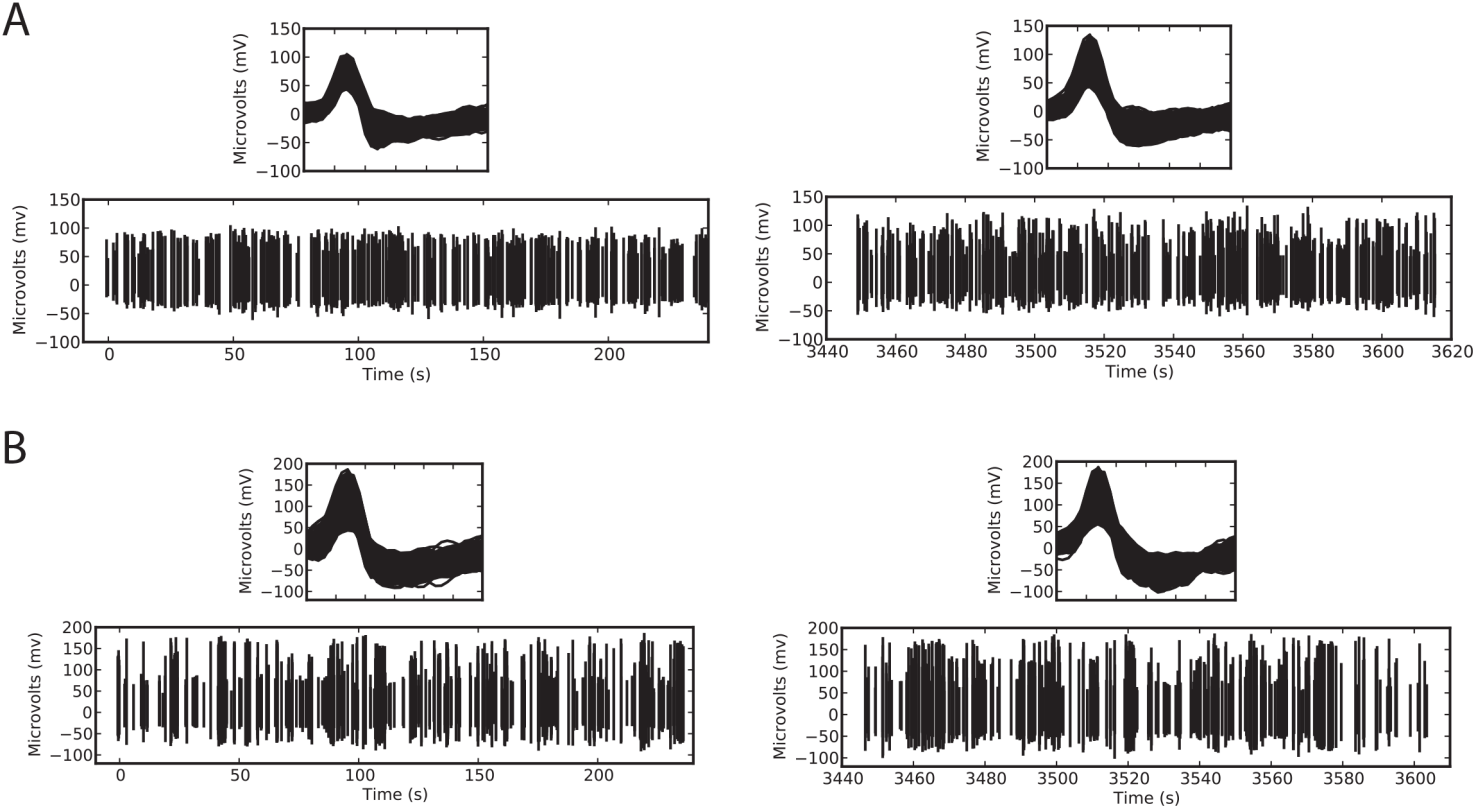
Comparison of extracellular recording using PriED and a similarly designed professionally machined microdrive. Recordings were made using 700–900 k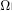 FHC tungsten electrodes. (A) Sample recording using the stacked base design and using a cannula to pass the electrode through the dura to the anterior cingulate cortex of an awake NHP. (B) Sample recording using a professionally machined version of the microdrive recording from the basal forebrain in an awake NHP. Both panels show two excerpts from the recordings: 500 action potentials from the start of recording (near time 0s) and 500 action potentials from the end of the session (around 3500s). The insets show details of the recorded action potentials.

Three groups in our lab have now been successfully using PriED to collect data single-neuronal data for about one year. We have found that the stability of the recordings is satisfactory. Importantly, advancing or retracting the electrode does not dislodge neurons isolated on other towers, even when the electrodes occupy adjacent grid holes. We have also found that subject movements do not typically cause us to lose isolation. As with all manual advancers one must keep manual track of the distance advanced by keeping count of turns. The practice in the lab is to keep a sheet indicating turns advanced and neurons encountered. Figure 2 shows typical samples of neural recording conducted using the stacked version of PriED and a similarly designed professionally machined microdrive system.

### Design Repository

A git based repository containing the current drive design has been setup at https://bitbucket.org/multidrive/pried. We hope that our positive experience with designing and manufacturing this 3-d printed recording device will inspire other researchers to download and print our design and then make modifications and share these modifications with other researchers, as we have done, via the repository.

## Discussion

We present an innovative micro-drive solution for flexible multisite cortical and subcortical recordings in the non-human primate. This solution utilizes a rapid prototyping manufacturing process commonly referred to as 3-d printing. By using this technology, researchers are able to create custom solutions to explore their experimental questions with unprecedented flexibility. In addition, by combining 3-d printed components with off-the-shelf components, we show that high quality and precise tools can be produced at a fraction of the cost it takes to manufacture these components in traditional model shops that require expensive hardware and experienced machinists.

### Tolerances of Dimensions

The bases need to fit snugly on the recording chambers but must not jam in them. This requirement is made more challenging by the fact that the chambers themselves have small variations in diameter. The current base dimensions (outer diameter of recording grid and inner diameter of the lip that goes over the chamber) were derived empirically over several iterations. The current dimensions work well with Crist chambers. However, we believe that the dimensions are sensitive to the printer and material used. Our experience is that outer diameters (e.g. of the grid) are slightly larger (∼0.4 mm) than designed and inner diameters (the lip) are smaller than designed - indicating that there are extra layers of material on all surfaces. When using a printer and material for the first time, we would suggest printing a prototype of the design to verify that the dimensions are acceptable. We expect that as the technology advances such issues will decrease and printed parts will conform more closely to the design dimensions.

### Printing of the Bases

The fineness of the base grids pose potential problems for 3-d printing. In our first iterations, where we utilized a 3-d printer in our model shop (Object 30, with a print resolution of 600×600×900 dpi and tolerance 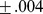), we found that while most parts printed satisfactorily, the printing of the base grid proved to be unreliable. In some runs the base came out distorted. This issue was completely solved by using a higher resolution printer from a commercial service.

### Future Directions

**Figure 3:**
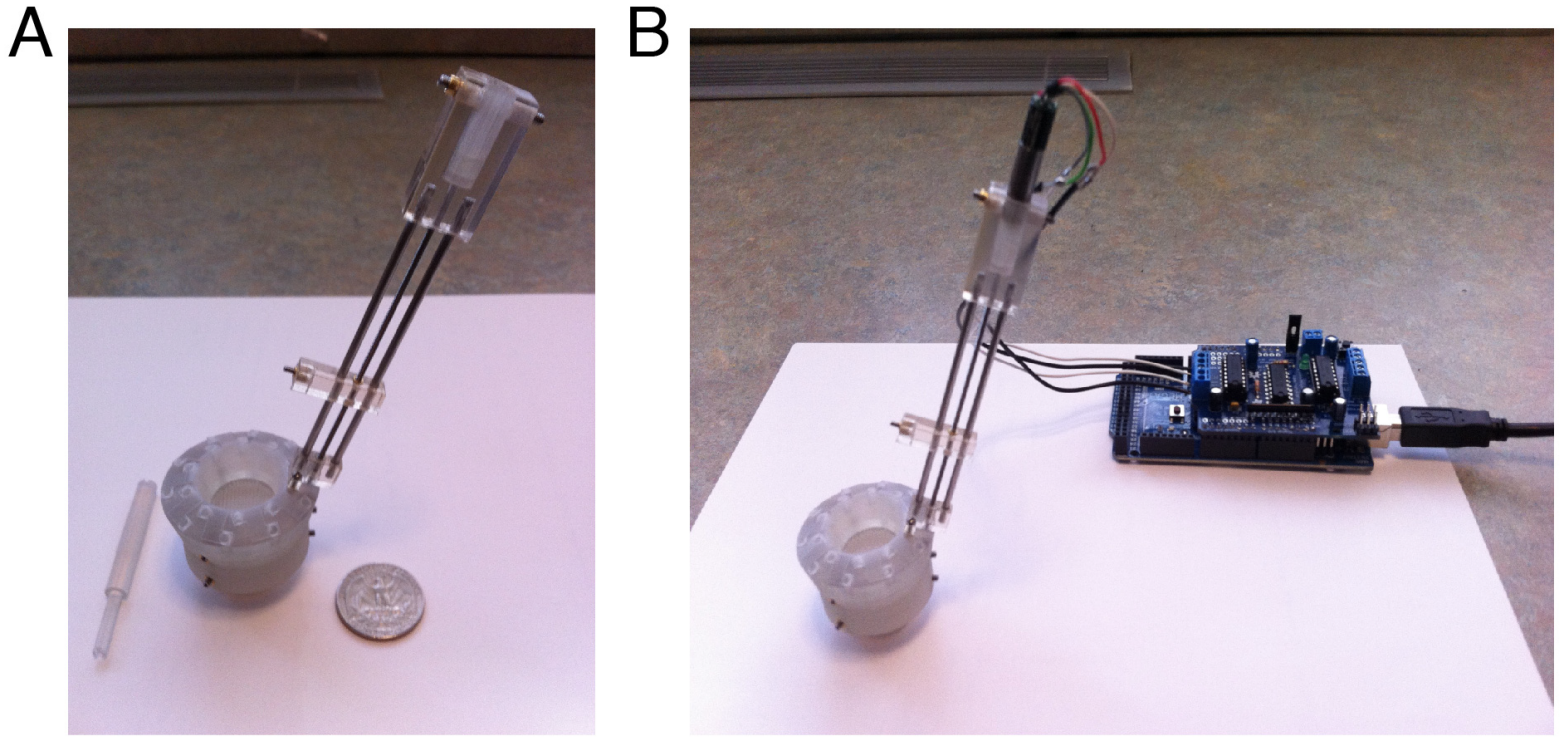
Photos displaying both manual and motorized PriED configurations. (A) Manual version with 3-d printed advancer. (B) Motorized version, showing stepper-motor, gear reduction box, and microcontroller. PriED is easily switched from manual to motorized configuration by removing the manual advancer and sleeve from the tower and sliding the motor in their place. The motor is then held in place by tightening the same set screw that formerly held the sleeve.

The next step for developing PriED is to incorporate a motor into the tower to drive the shuttle. The current device has been designed with this future extension in mind. The upper part of the towers are designed to accept a small stepper motor (Stepper Motor, Series ADM 0620, Faulhaber). The shaft of the stepper motor is fitted with a custom printed part (the upper adapter). The upper adapter is keyed to mate with the lower adapter just as the manual advancer is. The manual advancer and sleeve are simply removed and the motor is inserted in their place and secured with the same set-screw. The motor is controlled through an off-the-shelf micro-controller solution (an Arduino) with custom software and a hardware/computer controlled interface. The motorized version of PriED is shown in Figure 3.

We are currently testing the characteristics of this motorized drive. Two potential challenges to this solution are the small torque of the motor and the effect of the electromagnetic interference of the motor on the recordings. The issue of small torque is being addressed by using a reduction gear system (Planetary Gearhead, Series 06/1, Faulhaber) which has the added benefit of increasing the linear resolution of the drive. The issue of electromagnetic interference is potentially irrelevant since the interference only exists during motion and experiments are typically performed by advancing the electrode by small distances and then waiting to allow the tip and adjacent brain matter to settle while listening for neurons.

It is not clear to us that the commonly available 3-d print materials (such as the Vero series of plastics) are suitable for chronic implantation in a subject. However new materials for 3-d printing are becoming available that have been cleared for chronic implantation in humans (e.g. OsteoFab from OPM, CT, USA). This raises the exciting possibility that a low cost chronically implanted version of PriED can realistically be implemented with a year or two.

Perhaps more important than the flexible micro-drive solution itself, is the notion that these devices can be disseminated to a wide audience through a common online shared repository. A simple example is the ability to custom print angled grids to increase the access area afforded by a given chamber placement. A more elaborate example is the modification of the shuttle and grid design to hold glass pipette electrodes to perform iontophoresis of a pharmaceutical agent to one brain region while simultaneously recording single-neuronal activity from a downstream brain region. The opportunity to easily design and freely share and use the created micro-drive solutions should be a great boon to electrophysiologists, who are by nature tinkerers and improvisers as far as recording equipment goes.

## Materials and Methods

### Overview

**Figure 4:**
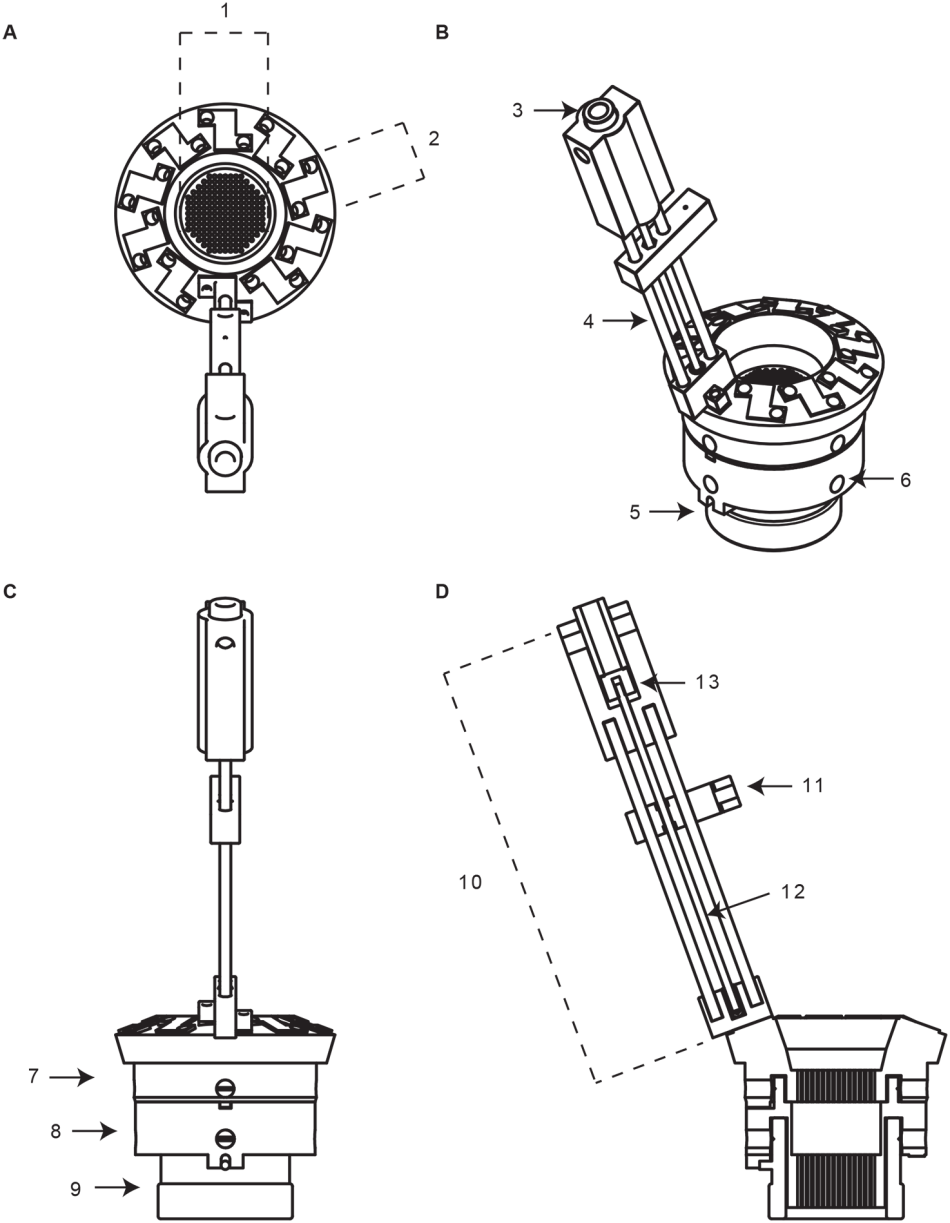
Schematic of complete PriED assembly, showing stacked base on chamber with one tower attached. (A) Top-down view of micro-drive highlighting the grid (1) and pad (2) where each tower attaches to the base. (B) Isometric view of the micro-drive. The sleeve (3), rails (4), notch that aligns with the grid (5), and set screw that attaches to the chamber (6). (C) Side-view highlighting the upper (7) and lower (8) base of the micro-drive when attached to the chamber (9). (D) Cut-away view showing tower (10), shuttle (11), lead screw (12), and upper adaptor (13).

**Figure 5:**
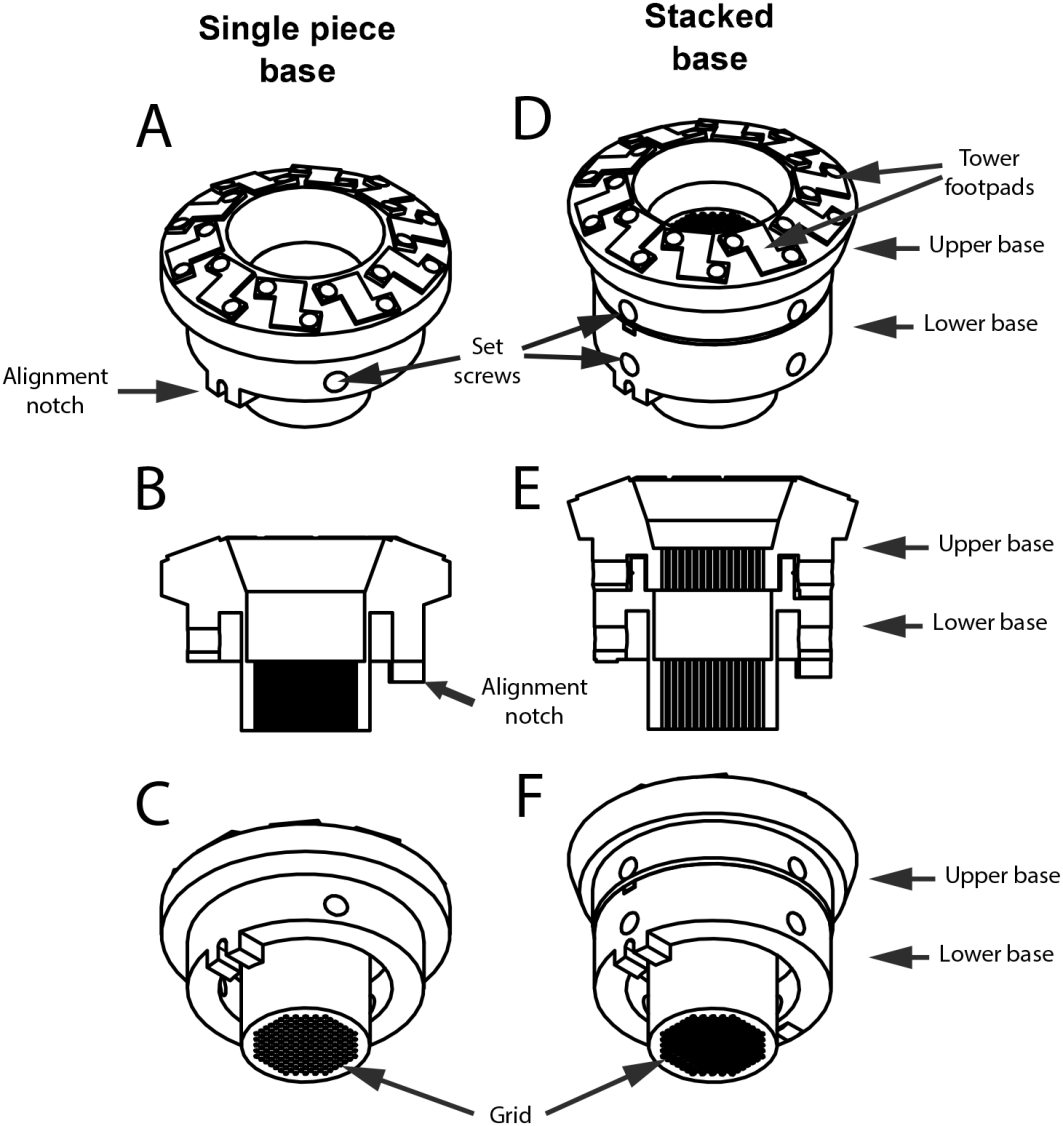
Schematics of single and stacked bases. (A,B,C) Single piece base shown from top-down isometric, sectioned and bottom-up isometric views. Similarly for (D,E,F) for the stacked base.

PriED consists of two main components: a base and one or more towers (Figure 4). The base is the component that mates and attaches to the recording chamber. We have designed and tested two variants of the base system. The first is a single-piece design that is compatible for use with dura-piercing electrodes (Figure 5A). The second is a stacked (two-piece) design that allows the use of cannula to guide electrodes to deeper brain structures (Figure 5B). Both base designs incorporate an alignment key and a 1 mm spaced grid that allow repeatable and precise electrode insertions. The bases are designed to attach to standard primate recording chambers (e.g. Crist Instruments) but can easily be modified to meet other requirements. The bases are designed to receive up to ten independently operated towers. The towers are angled such that all ten shuttles can descend to the bottom position without interfering with each other. This angled design was inspired by an earlier micro-drive developed in the lab with inspiration from microdrive systems used in rodent neurophysiology [16].

**Figure 6:**
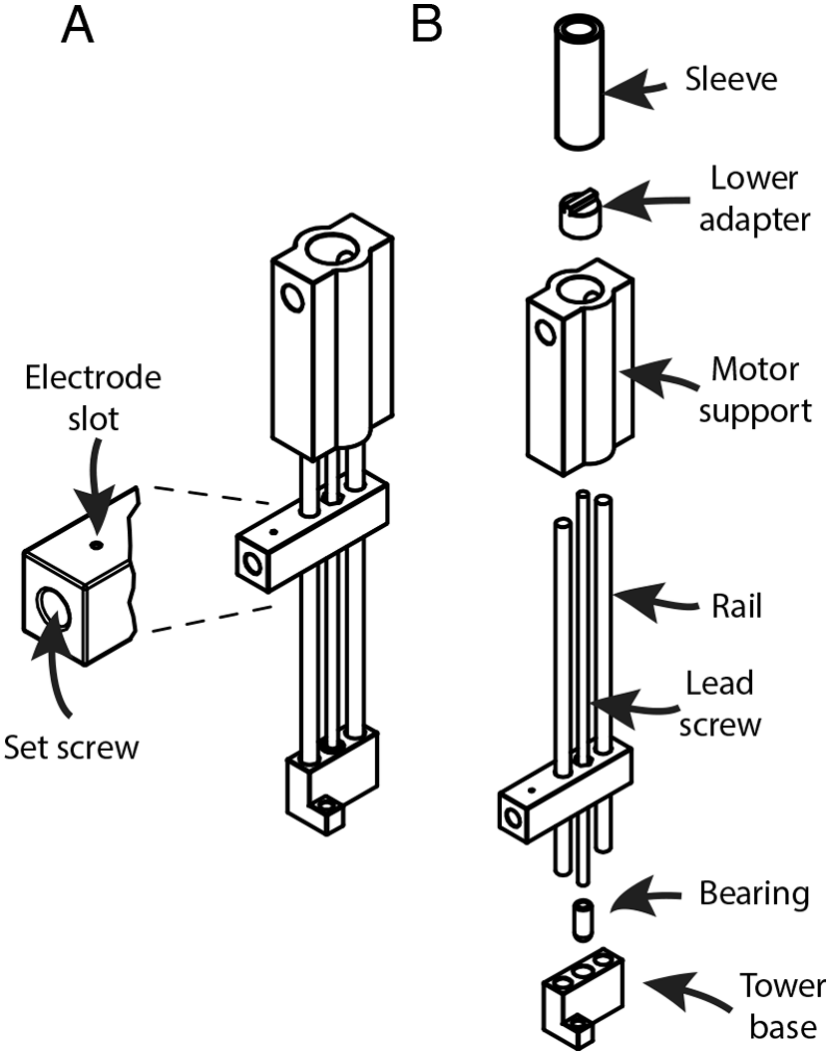
Schematic of electrode advancing tower assembly. (A) Complete tower assembly showing detail of shuttle that carries the electrode. (B) Exploded view showing tower components. From top to bottom, an assembled tower consists of: sleeve, lower-adapter, motor holder, rails and lead-screw, shuttle, bearing and tower base. Not shown are the two 0–80 nuts that press fit into the shuttle. The sleeve and the bearing hold the lead-screw assembly in place, preventing it from sliding along the tower. The manual advancer (not shown) is keyed to mate with the lower-adapter and is used to turn it, thereby rotating the lead-screw.

**Figure 7:**
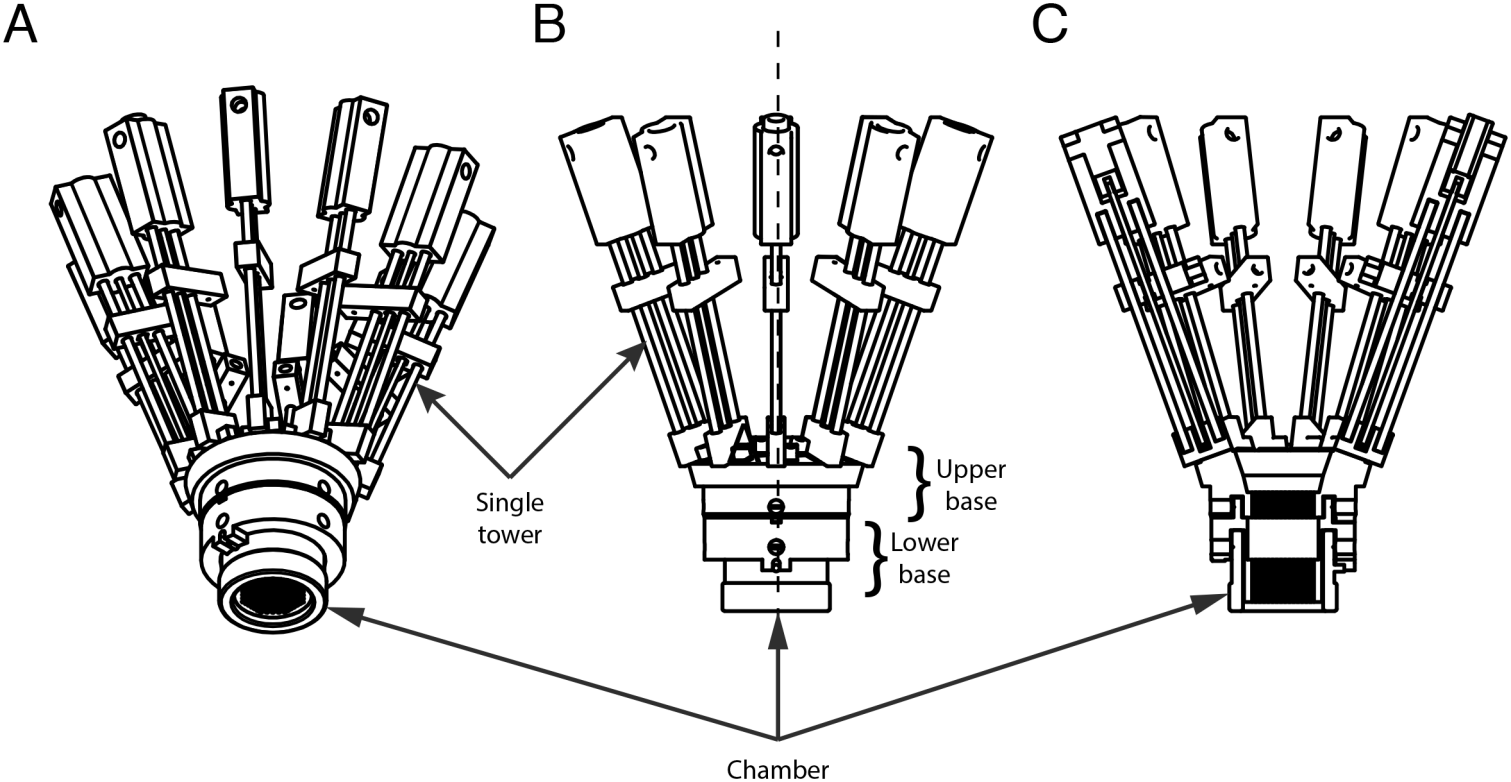
Schematic of stacked base with full complement of 10 towers. (A) Bottom-up, (B) Side view and (C) cross-section views. All towers are independent and can be attached in any spatial arrangement as convenient for the experiment.

The towers constitute the electrode advancing mechanism (Figure 6). Each tower consists of an upper and lower printed piece that hold two parallel metal rails rigidly in place. A threaded rod, centered between the two rails, serves as a lead screw. The lead screw is rotated using a manual advancer, driving an electrode shuttle that rides along the rails. The shuttle securely grips the electrode and allows for stable high resolution control of its movement. An off-the-shelf 0–80 threaded rod is used for the lead screw, giving a linear movement resolution of 300 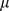
m/revolution. Each tower attaches to the base via two screws and bushings. The towers are independent of each other and up to 10 can be added to a base (Figure 7) and switched between bases as needed.

In the current design we took care to use simple, commercially available, metal parts where ever we expected wear and tear to occur, such as the set-screw housings and the nut that travels up and down the lead screw, propelling the shuttle. Though the use of metal parts adds some time to the assembly of the drive, it improves durability. The entire device is very easy to prepare and assemble. The metal parts mate with the plastic parts either through press fit or with glue.

The modular design of PriED and the use of 3-d printing for the parts allows researchers to independently customize the base and towers to meet the needs of their experiments. For example, in this paper we present two different designs for the base which allows the same towers to be used either with stiff dura-piercing electrodes or with thin electrodes guided into the brain via cannulae. Additionally, with simple editing of the designs using 3-d CAD software, researchers can further customize the bases, for example, by angling the grid to access different targets using the same chamber. The use of cut-to-length metal rods and lead screws for the towers enables researchers to build drives with different electrode travel lengths, without needing to change the design of the printed parts.

### Printed Parts

All CAD drawings were created using Autodesk Inventor Professional 2013 (San Rafael, CA) and are hosted at a publicly accessible repository with versioning control (https://bitbucket.org/multidrive/pried). All parts were printed by R&D Technologies Inc. (North Kingstown, RI) by sending them. STEP files via email for each individual part. The parts were printed on an Objet Connex 500 printer using PolyJet deposition printing. On this printer, a typical accuracy of 20–85 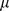
m can be expected for features under 50 mm and can print with a 16 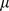
m layer thickness. The parts were printed using a translucent material called VeroClear FullCure 810, although a wide selection of materials are available with varying strength, color, and material properties.

#### A note on CAD software and file formats

In our opinion a major irritant in 3-d design is not the current state of the actual hardware and materials, which we anticipate will continue to improve rapidly with the popularization of the technology. Rather, the major issue is with the incompatible file formats used by different CAD software, with file formats changing destructively even between different versions of the same software. Fortunately, the industry has developed a standard for model description (ISO 10303, also known as STEP) which most printers can interpret. Another format, STL, is also commonly used with 3-d printing.

Unfortunately, while the data in the STEP files fully describe the model, they do not describe the constructive steps taken to build the model. This makes it harder to modify the model and make small changes quickly. For this reason, in our repository, we store STEP files (which conform to an open standard), STL files, as well as the files used by Autodesk Inventor natively. Though Autodesk is exemplary CAD software, it is proprietary and uses a proprietary file format. We would prefer if laboratories had access to open source CAD software. However we found that, in this case, the proprietary solution was much more reliable and easy to use than any open source or free software that we could find. It should be noted that Autodesk currently provides their software free of charge for non-commercial applications to academic institutions. It is our hope that with the popularization of 3-d printing such open source software will mature and become widely available. A promising candidate appears to be FreeCAD (http://free-cad.sourceforge.net/) though it is not quite usable in production yet.

### Off-the-shelf Metal Parts

Metal parts were sourced from McMaster-Carr (http://www.mcmaster.com/) and SmallParts (now part of AmazonSupply, http://www.amazonsupply.com/). A complete list of the metal parts needed, their function and their source and part numbers are given in Table 3.

**Table 3:** Bill-of-materials for off-the shelf metal parts.

Part description	Use	Source	ManufacturerPart number	Quantity
Brass press fitbushing (2–56)	Receives set screws for attaching bases	McMaster-Carr	92395A111	4/base
Brass press fitbushing (0–80)	Receives set screw to clampelectrode on shuttle and screwsto attach towers to base	McMaster-Carr	92395A109	3/tower
Cone point setscrew (2–56)	Set screw for attaching bases	McMaster-Carr	92785A055	4/base
Cup point setscrew (0–80)	Set screw for clampingelectrode on shuttle	McMaster-Carr	92311A055	1/tower
Phillips headmachine screw (0–80)	Secures towers to base	McMaster-Carr	91772A055	2/tower
Stainless steeltubing (14 G)	Rails	Small Parts^a^	h0083006t304who(B004WPPUKO^b^)	Cut to length
Threaded rod (0–80)	Lead screw	Small Parts	TRX-0080–24(B000FMWBNC^b^)	Cut to length
Brass nut (0–80)	Drives shuttle with leadscrew rotation	McMaster-Carr	92736A001	2/tower

a Now part of AmazonSupply, http://www.amazonsupply.com/.

b Part number on AmazonSupply.

#### Preparation of parts

All 3-d printing technologies use scaffolding material around the parts to help support the structure during the build. This, soft, loose material needs to be removed following the printing process. Our parts were printed and cleaned prior to delivery. However, we performed a second cleaning once we received the parts to ensure that all parts were clean prior to assembly. This includes verifying that all scaffolding material has been adequately removed around each piece, with particular attention to holes and the skirts of the bases. No special tools or chemical solutions were used.

### Assembly

There are a total of nine different printed plastic parts and four different kinds of metal parts. Prior to assembly, it is important to ensure that the plastic parts are free from debris that may have accumulated during the printing process. A minor amount of cleaning may be required for the plastic parts and some of the metal parts will need to be cut to length (for the tower assembly) depending on the requirements of the experiment. The assembly is made quick and easy by using press-fit or super glue gel (Loctite, Super Glue ULTRA Gel Control) to connect the plastic and metal parts. The towers are attached to the base using screws. The preparation of a base typically takes five minutes. The assembly of a tower typically takes twenty minutes.

#### Assembly of base

To assemble the base, either single or stacked, bushings need to be inserted into the four base holes. These bushings will hold set-screws that will either clamp the base to the chamber and/or the upper base to the lower base. To install, slightly crush the knurled end of a bushing using a pair of pliers so that it can easily fit into the hole. Once inside, take a set-screw and gently tighten it into the opening of the bushing. As the set screw enters into the bushing, it will expand the knurled face outwards into the plastic material forming a strong adhesion. Repeat this for each of the base holes. This same procedure will be used to install all the bushings throughout the PriED. The 0–80 bushings need to be installed in the screw holes on the top surface of the base that will receive the 0–80 screws that secure the towers to the base.

#### Assembly of tower

Each tower is composed of: one tower base, one motor support, two rails, one lead screw, one shuttle, one sleeve, and one bearing. Step-by-step instructions to assemble a tower are presented in Table 4. Tower assembly requires preparing two rails and one lead screw for each tower. The height of the tower depends on the length of the rails and lead screw. The researcher must determine the maxmum electrode travel length required to reach each target structure prior to cutting the rails and lead screw. We have used travel lengths of 70 mm to allow access to deep-brain structures such as the nucleus accumbens and the nucleus basalis. The travel length is the length of the rails and lead screw that the shuttle can travel; this does not include the portion that extends either into the tower base or motor support. The formulae in Eq. 1 and 2 should be used to calculate the rail and lead-screw lengths to cut from the stock material for a tower with electrode travel length *x_travel_*. The formulae account for the insertion of the rails into the end-pieces of the tower and the slightly longer length of the lead-screw, compared to the rails.
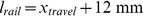
(1)

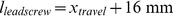
(2)


**Table 4:** Instructions to assemble a tower.

Step	Description
1	Clean out motor holder set-screw holes and insert 2–56 bushings. Expand them to grip
2	Cut rails and threaded rod to desired length
3	Press fit two 0–80 nuts into nut housings in shuttle
4	Screw threaded rod into a 0–80 nut a few times to even out threads on rod ends
5	Screw threaded rod through nuts embedded in shuttle
6	Attach bearing to the lower end of the lead screw
7	Assemble rails into lower base, slide shuttle onto rails
8	Assemble motor support onto rails and verify good fit
9	Slide threaded rod up to expose top end, screw on lower adapter to threaded rod
10	Slide rails off lower base, put a drop of super glue gel into the two rail wells, slide rails back into wells and wait till firm
11	Slide motor adapter off, add a drop of super glue gel into the two wells, flip tower upside down, insert rails into wells and wait till firm
12	Slide lead screw down till bearing sits in bearing well
13	Slide sleeve into motor housing and secure with set-screw

#### Assembly of drive

**Figure 8:**
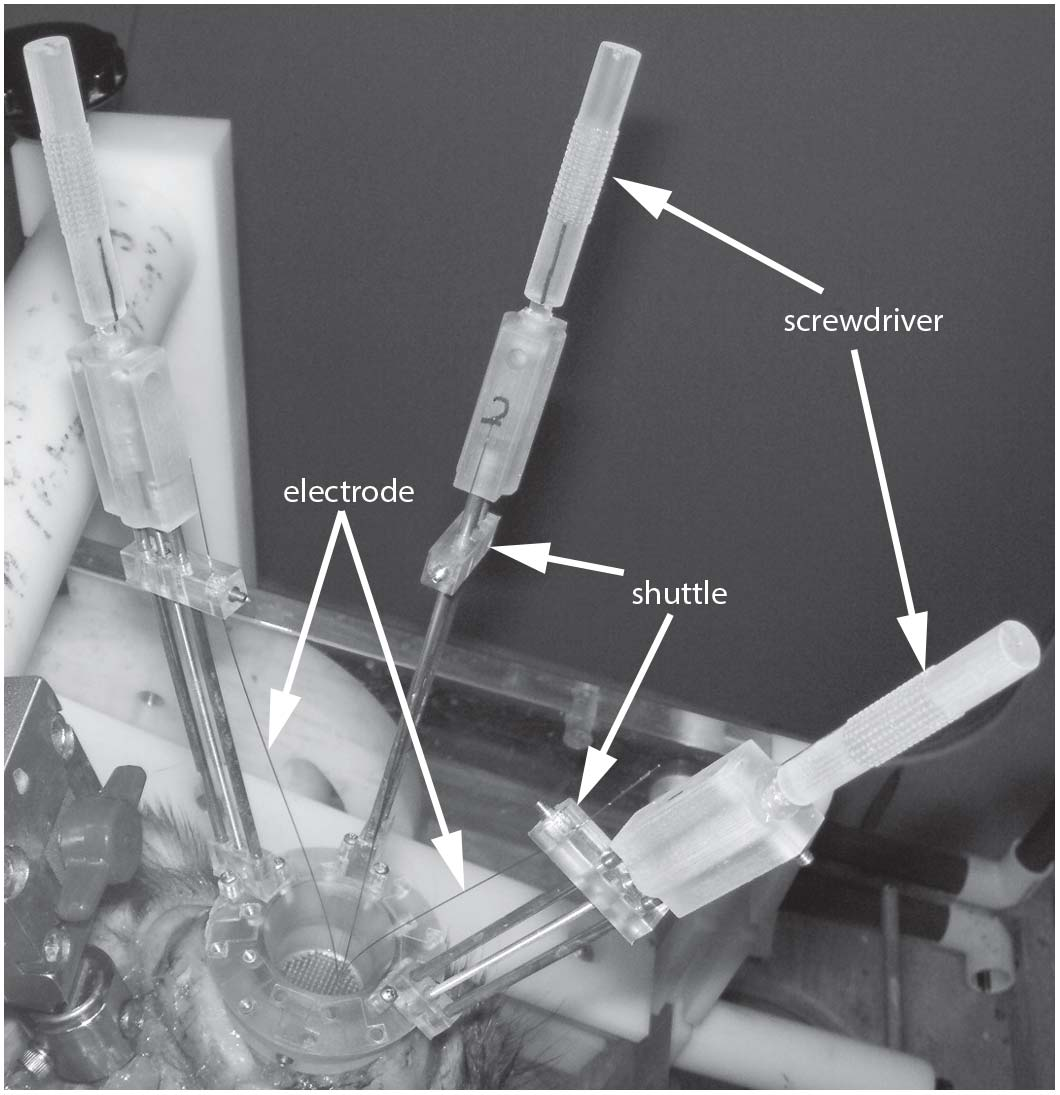
PriED in use. (A) The electrode drive is shown with the single-piece base and three towers in the fully retracted position at the start of a recording session. The towers are carrying metal-in-glass electrodes that are positioned in three adjacent grid holes. The tower heights allow for 70 mm electrode travel which enables the towers to be used for deep brain recordings. The manual advancers are seen protruding from the tops of the towers.

Once the towers and base are prepared it is a simple matter to use the 0–80 Philips head screws to secure each tower to the base in the desired configuration. Figure 8 shows an assembled drive with single piece base and three towers ready for recording.

### Zeroing the Drive for Recording

For the single base, the recommended method for standardizing the electrode start position is to load the electrode flush to the bottom of the lower grid. For the stacked base, load the electrode flush to the bottom of the upper grid. It is important to note that when using the stacked base, an additional 20 mm of dead space is created, and therefore electrode lengths should be appropriately adjusted to ensure travel to target structures.

### Preparation and use of Cannulae with the Stacked Base

**Figure 9:**
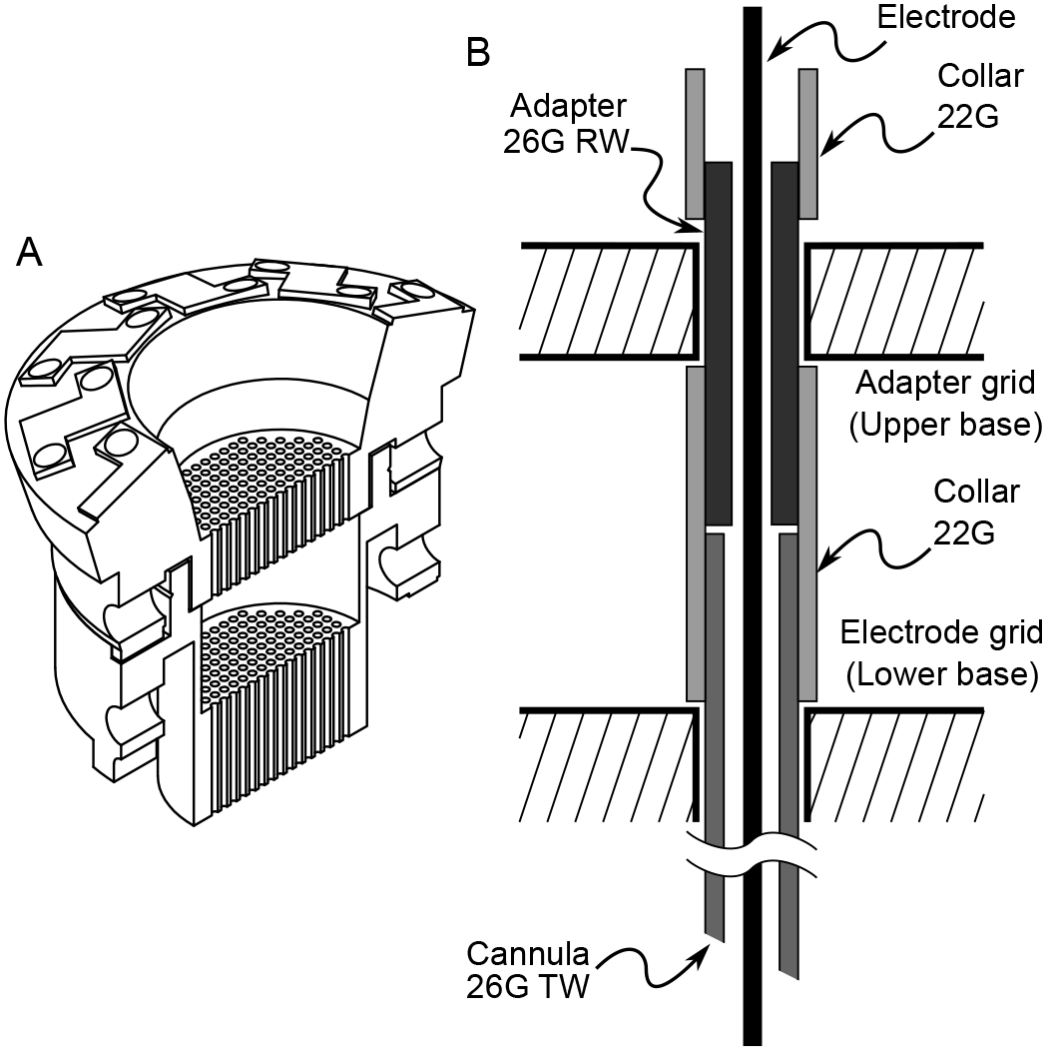
Stacked base and cannula system. (A) The stacked base is printed as two parts. The lower base (electrode grid) attaches to the recording chamber and allows positioning of the cannula. The grid holes are 500 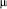
m in diameter to accommodate 26G cannuli. The upper base (adapter grid) recieves the towers, holds the electrode adapters and mates with the electrode grid after the cannuli are in place. (B) The cannula is cut from 26G TW (thin wall) tubing with 22G tubing as collar. The adapter is cut from 26G RW (regular wall) tubing. The slightly smaller inner diameter of the adapter compared with the cannula reduces the likelihood of electrode damage as the electrode is advanced.

The two-piece, stacked base is designed for experiments that use cannulae to puncture dura. The use of cannulae allows for the use of tetrodes or fine electrodes which would otherwise be damaged when passing through dura. The use of cannulae with the stacked base configuration is illustrated in Figure 9. The use of the adapter ensures that the electrode can be advanced from its zero position down into the canula without damaging its tip.

#### Cannula and adapter

The gap between the lower grid and the upper grid is 10 mm. The cannula collar must therefore be slightly less than this clearance. The cannula itself should extend midway into the collar (5 mm) leaving an equal distance for the adapter to slide in and collimate satisfactorily, allowing the electrode to pass into the cannula without tip damage. The adapter itself should be fitted with a collar to prevent it from slipping out of the upper grid. When mated with the cannula, the adapter should not protrude above the rim of the upper base, otherwise it will reduce electrode travel by interfering with the shuttle.

The design of the stacked base is compatible with 26G cannulae. The cannulae themselves are cut from 26G TW (thin walled) hypodermic tubing. The electrode adapter that mates with the cannulae are cut from 26G RW (regular walled) hypodermic tubing. Because the inner diameter of the adapter is smaller than that of the cannula, there is much less chance of the electrode tip hitting the side of the cannula as the electrode slides in.

#### Recording with cannulae

The electrode grid (lower base) and the adapter grid (upper base) are readied separately. An adapter is placed in the desired grid position and an electrode is back-loaded through the adapter and attached to the shuttle. The adapter is pulled up, flush with the adapter grid bottom and the electrode position is zeroed as described previously. A cannula with its protective stylette is inserted into the corresponding grid hole in the lower base.

To begin recording, the lower base is secured to the recording chamber. The cannula is then passed through the dura and the stylette is removed. The upper base is mated with the lower base and secured. The adapter is then pushed down until it is firmly mated with the cannula (The mated configuration is shown in Figure 9). The electrode is then advanced down into the brain.

### Maintenance and Cleaning of Drive

We found that the finer parts made of the VeroClear material, such as the grid, can be damaged by even short exposure to alcohol and to acetone. We have found that it is best to rinse out the grid and cannulae with Nolvasan (Chlorhexidine solution) followed by another wash with distilled water, both before and after recording sessions. In our experience, this was sufficient for maintaining the cleanliness of the system without compromising structural integrity over time. It is also best to store the drive in a drawer or box to prevent dirt from accumulating on the threads of the lead screw.
